# Supplementary material for: Radiative plasma simulations of black hole accretion flow coronae in the hard and soft states
Source: Nat Commun. 2024 Aug 15;15:7026. doi: 10.1038/s41467-024-51257-1 (PMC11327351; doi:10.1038/s41467-024-51257-1)
Supplement: Supplementary file 1 — Supplementary Info [file 41467_2024_51257_MOESM1_ESM.pdf]

Radiative Plasma Simulations of Black Hole Accretion Flow Coronae in the Hard and Soft States

Joonas Nättilä[1,2,3*]

[1]Department of Physics, University of Helsinki, Gustaf Hällströmin katu 2, Helsinki, 00014, FI, Finland.
[2]Columbia Astrophysics Laboratory, Columbia University, 538 West 120th Street, New York, 10027, NY, USA.
[3]Center for Computational Astrophysics, Flatiron Institute, 162 Fifth Avenue, New York, 10010, NY, USA.

Corresponding author(s). E-mail(s): joonas.nattila@helsinki.fi;

Supplementary Methods

The numerical parameters and simulation techniques are detailed here. We simulate the radiative plasma dynamics with RUNKO framework [1]. The QED module, which is responsible for the radiative processes, includes the exact differential cross-sections of each process and solves them with a Monte Carlo sampling method.

Our fiducial numerical simulation setup employs a 640^3 grid with a physical box size of $l_0 = 640\, c/\omega_{\rm p}$. We have found that a minimum box size that exhibits converged dynamics behavior has $l_0 \approx 300 c/\omega_{\rm p}$. In addition, we have tested that similar results are produced by larger boxes of 1280^3 (with $l_0 = 1280\, c/\omega_{\rm p}$) and with higher skin depth resolution. Simulation domains with $l_0 \lesssim 300\, c/\omega_p$ (or about 300^3) have too limited turbulence inertial range to have realistic intermittent energization regions.

The discussed simulations are designed to minimize the memory footprint (and to maximize the domain size); in practice, this means minimizing the number of computational particles while accurately capturing the plasma physics. We resolve the plasma momentum distributions with ≈ 10 computational particles per cell per species (ppc). We have verified that a particle resolution of ≈ 5 ppc is enough to capture the large-scale plasma phenomena (mainly to provide a smooth charge for the electric currents). In addition, we smooth the electric current before deposition with 6 binomial filter

passes. We have compared these simulations to shorter simulations with higher ppc values (up to 32) and found the combination of 10 ppc and 6 filter passes to mimic these higher resolution runs.

Energy is continuously injected into the domain with an oscillating Langevin antenna that excites 8 plane-wave modes on a scale l_0. We have found the resulting turbulence to be largely insensitive to the details of the driving: there is no strong dependency on the driving amplitude (for $0.5 \lesssim \delta B/B_0 \lesssim 2$), antenna oscillation frequency (for $0.5 \lesssim \omega_{\rm ant}/\omega_0 \lesssim 1$), or mode decorrelation frequency (for $0.5 \lesssim \omega_{\rm dec}/\omega_{\rm ant} \lesssim 1$). In fact, previous studies seem to indicate that there is even little difference in plasma energization mechanisms seen in controlled numerical experiments between decaying and driven energy injection if the computational box is large enough [e.g., 2, 3].

We resolve the QED reactions on a coarse-grained grid of 64^3 "tiles"; each tile has a side length of $h_{\rm rad} = 10c/\omega_{\rm p}$ (i.e. 10^3 grid points). Such coarse-graining is required to combat the Monte Carlo sampling noise; however, the technique is also physically motivated since photons have a longer mean-free path between interactions, so a larger effective sampling region is justified. The reported simulation includes all of the main radiative processes [4]: Compton scattering, synchrotron radiation, synchrotron self-absorption (SSA), two-photon pair creation, and pair annihilation. All processes, except SSA, are simulated using binary Monte Carlo interactions. We have validated our radiative process implementations against the extensive tests described in [5, 6].

We simulate SSA with a special hybrid technique where the local radiation field in the tile is first mapped onto a grid, and the SSA opacity is then calculated [5]. The resulting opacity is used in a single-particle interaction where a (low-energy) photon has a finite chance of being absorbed or an electron/positron has a finite chance of being thermalized. The method is tested to reproduce the expected SSA photon spectrum and electron heating rate. In practice, the reported simulations are insensitive to the details of the SSA implementation; the main effect of SSA is to function as a quasi-thermal photon source with a temperature comparable to $x_{\rm SSA} \approx 20 x_{\rm syn}$ [7] and a luminosity $\ell_{\rm SSA} \approx 0.1$ (given the simulated parameter regime).

We enhance the photon statistics—while simultaneously aiming to minimize the simulation memory load—by employing adaptive photon weights in the simulations. We keep the electron/positron weights fixed to avoid numerical heating. We have found this usage of adaptive Monte Carlo technique [5] crucial for accurately capturing the two-photon pair creation process rate—high-energy photons at the tail of the distribution drive the process with $x \gtrsim 1$. We have found that the physically correct simulations (i.e., correct pair-creation rates) require on the order of 100 photon particles per cell. We tune the photon weight splitting and merging rates so that we have ≈ 200 photons per cell at any given time, with 70% of the photons with energies $x \gtrsim 0.1$: in practice, we use an empirical particle weighting function of $f(x) = (x/0.01)^{0.04}$ as described in Ref.[5].

We induce the phase transition in the system (between hard and soft states) by injecting additional soft photons into the domain. These extra photons are designed to mimic the radiation from the optically-thick inner disk. A real disk emission would be a

multi-temperature black body function; however, for simplicity, we model the external photon as a single-temperature black body with a temperature of $k_\mathrm{B}T_\mathrm{ext} \approx 0.3\,\mathrm{keV}$ and a varying luminosity ℓ_ext. In practice, we inject each tile with $\zeta_\mathrm{ext} = 0.001 N_x$ thermal photons every time step, where N_x is the number of photons in the tile (typically $N_x \sim 10^6$). The injection is homogeneous and is done independently on each tile. The homogeneous injection remains a valid approximation for low-luminosity systems ($\ell \lesssim 100$) since the mean free path of the photon needs to exceed the tile length for it to be valid.

We model the photon escape from the domain with an escape probability formalism [e.g., 5]. Here, each photon has a probability rate of $p_\mathrm{esc} = -\dot{n}_x/n_x$ for being removed from the domain, where $\dot{n}_x \equiv \mathrm{d}n_x/\mathrm{d}t$, and n_x is the photon number density. We approximate the geometry of the domain as an infinite homogeneous slab of height H. In this case, the photon escape probability rate asymptotes to $p_\mathrm{esc} \to c/H$ for $\tau \ll 1$ and to $p_\mathrm{esc} \to c/H\tau$ for $\tau \gg 1$. We model the general solution as

$$p_\mathrm{esc} = \frac{c}{H}\frac{1}{1+\langle\tau\rangle f}, \qquad (1)$$

where $\langle\tau\rangle$ is the mean (Thomson) optical depth in the tile, and $f(x)$ is an empirical weighting function that accounts for the fact that as the photon energy increases, forward scatterings dominate, leading to a reduction in escape time [e.g., 5]. To the accuracy described here, the simulation results are insensitive to the details of the described formalism (e.g., [6]).

Supplementary Discussion

Here, we discuss the role of each QED process in regulating the system state. In addition, we outline the seed-photon generation mechanisms in the magnetized corona. Lastly, we also list the details needed to compare the simulations to real observations.

For illustrative purposes, we solve simplified kinetic equations describing the time evolution of the plasma and radiation number densities per momentum or energy interval, $n_\pm \mathrm{d}p$ and $n_x \mathrm{d}x$, respectively, where $p \equiv \gamma\beta$ is the (dimensionless) plasma momenta, γ is the Lorentz factor, β is the 3-velocity, and $x \equiv h\nu/m_e c^2$ the photon energy. We use a similar kinetic solver as in Ref. [8] and include the same QED processes as in our primary plasma simulations: Compton scattering (Compton), cyclo-synchrotron emission (synch), synchrotron self-absorption (SSA), pair annihilation/photon production (pa), and pair production/photon annihilation (pp). The coupled equations for n_x and $n_\pm$ are then [e.g., 9]

$$\begin{aligned} \frac{\partial n_x(x)}{\partial t} &= -\frac{n_x(x)}{t_\mathrm{x,esc}(x)} + \sum_{i\,\in\mathrm{processes}} \dot{n}_{x,i}(x) \\ \frac{\partial n_\pm(p)}{\partial t} &= -\frac{n_\pm(p)}{t_{\pm,\mathrm{esc}}(p)} + \sum_{i\,\in\mathrm{processes}} \dot{n}_{\pm,i}(p) + Q_\pm(p)\,, \end{aligned} \qquad (2)$$

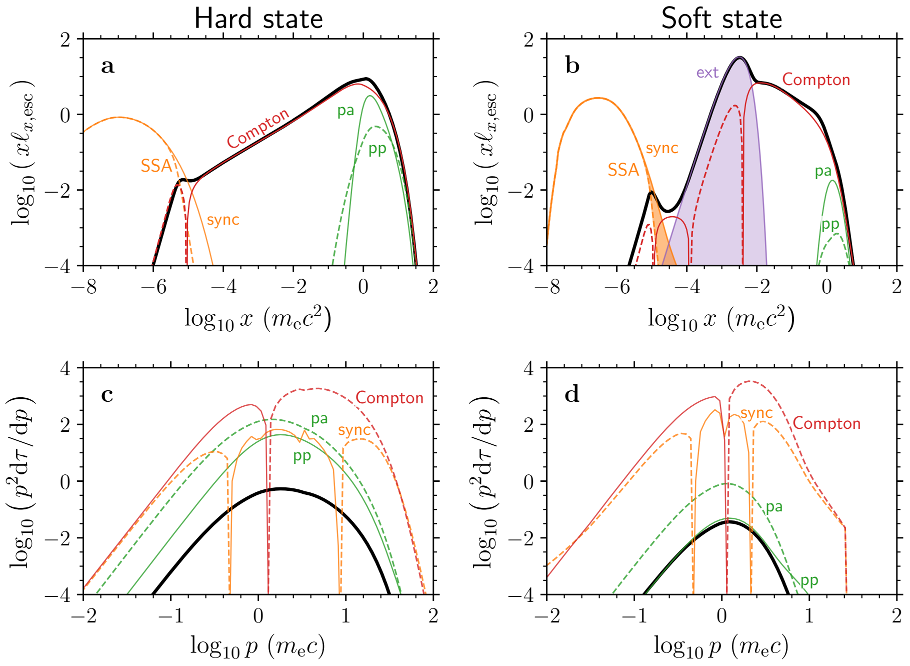

Supplementary Figure 1 Schematic view of the QED processes that control the state of the corona. a Spectra of the escaping radiation for a configuration mimicking the simulated hard state. The contribution of each process is shown with thin colored curves: processes with net gain ($\dot{n}_\mathrm{proc} > 0$) are shown with solid curves and net losses ($\dot{n}_\mathrm{proc} < 0$) with dashed curves. We include synchrotron self-absorption (denoted with SSA), synchrotron emission (synch), Compton scattering (Compton), pair annihilation (pa), pair production (pp), and external photon source (ext). Sources of the seed photons are highlighted with shaded regions. **b** Same visualization for the soft state. **c** The corresponding $e^\pm$ momentum distribution (in the units of the optical depth τ_T) that generates the radiation in the hard state. Thin lines show the effect of the QED processes on shaping the plasma distribution. **d** Same visualization for the soft state. The spectra are provided as Source Data files.

where the sum is over all the above-mentioned processes, $t_{x,\mathrm{esc}} \approx (1+\tau_\mathrm{T})H/c$ is the photon escape time, $t_{\pm,\mathrm{esc}}$ is the electron escape time, and $Q_\pm$ is an energy injection function. We solve for the steady-state of Eqs. 2 with $\partial n_{x/\pm}/\partial t \to 0$. Unlike in the full PIC simulations, here we have to devise an ad hoc heating function $Q_\pm(p)$ for the system. We choose to heat the plasma with a generic stochastic energization process following Ref. [9]. In addition, we inject non-thermal particles into the system with a power-law function $\mathrm{d}n_\pm/\mathrm{d}\gamma\mathrm{d}t \propto \gamma^{-\alpha_\mathrm{inj}}$ between $3 < \gamma < 30$. By studying such a simplified system, we can more easily demonstrate the role of each QED process in our full PIC simulations by considering the pair/photon production rates for each process $\dot{n}_{\pm,x}$; these are visualized in Supplementary Figure 1.

We model the hard state (see Supplementary Figure 1a and c) with a slab of size $R = 3\times10^7$ cm and height of $H = 0.3R$ so that its total volume is HR^2. The diffusive heating power (expressed via the dimensionless compactness parameter) $\ell_\mathrm{th} = 7$, non-thermal injection power $\ell_\mathrm{nth} = 1$, injection slope $\alpha_\mathrm{inj} = 6$, and magnetic compactness $\ell_B = 0.1(\ell_\mathrm{th} + \ell_\mathrm{nth})$. We set $t_{\pm,\mathrm{esc}} \to \infty$. These parameters result in a similar $n_\pm$

and n_x as in our full radiative/QED PIC simulations: the plasma (Thomson) optical depth is $\tau_\mathrm{T} \equiv H\sigma_\mathrm{T} \int n_\pm(p)\mathrm{d}p \approx 3$ and the escaping radiation flux is on the order of $10^{36}\,\mathrm{erg\,s^{-1}}$. We note that the escaping radiation spectra peak at a higher energy ($x \approx 1$) than the resulting photon spectra from the full radiative/QED simulation (with the peak at $x \approx 0.2$) because here the radiation is up-scattered by the regular Compton interactions between the hot plasma—in the first-principles PIC simulations the up-scattering is dominated by the turbulent bulk Comptonization [10].

Similar to the hard-state PIC simulations, the seed photons originate from the cyclo-synchrotron emission that overcomes the SSA at about $x_\mathrm{SSA} \approx 10^{-5}$ (see the highlighted region in the Supplementary Figure 1a). These seed photons are then Compton up-scattered to $x \sim 1$ by the hot plasma (or the bulk Comptonization in the PIC simulations). The photon annihilation suppresses the high energy tail at $x \gtrsim 1$. The plasma distribution, on the other hand, is controlled mostly by the Compton scattering; notably, cooling the high-energy tail at $p \gtrsim 1$ occurs via Compton losses. The first-principles simulations broadly agree with this picture.

Next, we model the soft state (see Supplementary Figure 1b and d) with a cube of size $H = 3 \times 10^7$ cm. We set $\ell_\mathrm{th} = 5$, $\ell_\mathrm{nth} = 5$, $\alpha_\mathrm{inj} = 6$, and $\ell_B = 1(\ell_\mathrm{th} + \ell_\mathrm{nth})$. In addition, the disk emission is modeled with an external photon black-body distribution (EXT) with a temperature $k_\mathrm{B}T_\mathrm{bb} = 8 \times 10^{-4} m_e c^2$ and injection power $\ell_\mathrm{ext} = 15$. We set the plasma escape time $t_{\pm,\mathrm{esc}}$ so that the optical depth of the system $\tau_\mathrm{T} \approx 0.5$. Again, these parameters result in plasma and radiation distributions similar to the PIC simulations.

Similar to the full soft-state PIC simulations, the radiation spectra are dominated by the external black-body distribution that escapes through the optically thin plasma. Unlike in the hard state, here the photons originate from both the high-energy part of the cyclo-synchrotron emission and from the external photon distribution (see the highlighted regions in Supplementary Figure 1b). Like the full PIC simulations, the state transition from a hard-state-like spectrum to soft-state-like spectra is triggered by the dominant seed photon source changing from the SSA photons to the external photons.

We emphasize that while the parameters used here result in spectra that resemble the Cyg X-1 observations, they are still selected ad hoc. In the main text, we demonstrate with full radiative/QED PIC simulations that these parameter values originate from first-principles modeling of the turbulent plasma and the in-situ-calculated radiation field. Moreover, intermittent energization by plasma turbulence is a natural microphysical mechanism for sustaining the required hybrid plasma distribution with heating and non-thermal acceleration.

Lastly, we note that the simulated radiation output should be properly processed before it can be compared to real X-ray observations. We compare our simulations to Cyg X-1 observations as analyzed in [11]. To provide a fair comparison to the data, we attenuate the simulated spectra with a photoelectric absorption at $\lesssim 1\,\mathrm{keV}$ and add a Compton reflection component from the disk in the range of about $5-20\,\mathrm{keV}$. The photo-absorption is modeled using the PHABS model (with column density $N_H = 4 \times 10^{21}\,\mathrm{cm^{-2}}$; [12]) and Compton reflection using the REFLECT model (with solid angle $\Omega/(2\pi) \approx 0.2$ and ionization parameter $\zeta = 0$; [13]).

References

[1] Nättilä, J. Runko: Modern multiphysics toolbox for plasma simulations. A&A **664**, A68 (2022).

[2] Zhdankin, V., Werner, G. R., Uzdensky, D. A. & Begelman, M. C. Kinetic Turbulence in Relativistic Plasma: From Thermal Bath to Nonthermal Continuum. Phys. Rev. Lett. **118**, 055103 (2017).

[3] Comisso, L. & Sironi, L. Particle Acceleration in Relativistic Plasma Turbulence. Phys. Rev. Lett. **121**, 255101 (2018).

[4] Rybicki, G. B. & Lightman, A. P. Radiative Processes in Astrophysics (1986).

[5] Stern, B. E., Begelman, M. C., Sikora, M. & Svensson, R. A large-particle monte carlo code for simulating non-linear high-energy processes near compact objects. MNRAS **272**, 291–307 (1995).

[6] Coppi, P. S. Poutanen, J. & Svensson, R. (eds) The physics of hybrid thermal/non-thermal plasmas. (eds Poutanen, J. & Svensson, R.) High Energy Processes in Accreting Black Holes, Vol. 161 of Astronomical Society of the Pacific Conference Series, 375 (1999). astro-ph/9903158.

[7] Vurm, I., Lyubarsky, Y. & Piran, T. On Thermalization in Gamma-Ray Burst Jets and the Peak Energies of Photospheric Spectra. ApJ **764**, 143 (2013).

[8] Poutanen, J. & Vurm, I. On the origin of spectral states in accreting black holes. ApJ **690**, L97–l100 (2009).

[9] Vurm, I. & Poutanen, J. Time-dependent modeling of radiative processes in hot magnetized plasmas. ApJ **698**, 293–316 (2009).

[10] Grošelj, D., Hakobyan, H., Beloborodov, A. M., Sironi, L. & Philippov, A. Radiative Particle-in-Cell Simulations of Turbulent Comptonization in Magnetized Black-Hole Coronae. Phys. Rev. Lett. **132**, 085202 (2024).

[11] Zdziarski, A. A., Poutanen, J., Paciesas, W. S. & Wen, L. Understanding the long-term spectral variability of cygnus x-1 with burst and transient source experiment and all-sky monitor observations. ApJ **578**, 357–373 (2002).

[12] Balucinska-Church, M. & McCammon, D. Photoelectric Absorption Cross Sections with Variable Abundances. ApJ **400**, 699 (1992).

[13] Magdziarz, P. & Zdziarski, A. A. Angle-dependent Compton reflection of X-rays and gamma-rays. MNRAS **273**, 837–848 (1995).
